# Diagnostic value of 3D volume measurement of central pulmonary artery based on CTPA images in the pulmonary hypertension

**DOI:** 10.1186/s12880-023-01180-6

**Published:** 2023-12-13

**Authors:** Wanwan Zhao, Jialing Guo, Ningli Dong, Huanhuan Hei, Xiaoyi Duan, Cong Shen

**Affiliations:** 1 Department of PET/CT, The First Affiliated Hospital of Xi’an Jiaotong University, 277 Yanta West Road, Xi’an, Shaanxi 710061 China; 2 Department of Imaging, Nuclear Industry 215 Hospital of Shaanxi Province, Xianyang, Shaanxi 712200 China; 3 Department of Ultrasound, Jingbian People’s Hospital, Yulin, Shaanxi 718500 China; 4 Department of Imaging, The Second Hospital of Traditional Chinese Medicine of Baoji, Baoji, Shaanxi 721300 China; 5 Department of Imaging, The Second Hospital of Xi’an Medical College, Xi’an, Shaanxi 710038 China

**Keywords:** Pulmonary hypertension, Computed tomography pulmonary angiography, right cardiac catheterization, central pulmonary artery, Volume measurement

## Abstract

**Background:**

This retrospective study aims to evaluate the diagnostic value of volume measurement of central pulmonary arteries using computer tomography pulmonary angiography (CTPA) for predicting pulmonary hypertension (PH).

**Methods:**

A total of 59 patients in our hospital from November 2013 to April 2021 who underwent both right cardiac catheterization (RHC) and CTPA examination were included. Systolic pulmonary artery pressure (SPAP), mean PAP (mPAP), and diastolic PAP (DPAP) were acquired from RHC testing. Patients were divided into the non-PH group (18 cases) and the PH group (41 cases). The diameters of the main pulmonary artery (D_MPA_), right pulmonary artery (D_RPA_), and left pulmonary artery (D_LPA_) were measured manually. A 3D model software was used for the segmentation of central pulmonary arteries. The cross-sectional areas (A_MPA_, A_RPA_, A_LPA_) and the volumes (V_MPA_, V_RPA_, V_LPA_) were calculated. Measurements of the pulmonary arteries derived from CTPA images were compared between the two groups, and correlated with the parameters of RHC testing. ROC curves and decision curve analysis (DCA) were used to evaluate the benefit of the three-dimensional CTPA parameters for predicting PH. A multiple linear regression model with a forward-step approach was adopted to integrate all statistically significant CTPA parameters for PH prediction.

**Results:**

All parameters (D_MPA_, D_RPA_, D_LPA_, A_MPA_, A_RPA_, A_LPA,_ V_MPA_, V_RPA_, and V_LPA_) of CTPA images exhibited significantly elevated in the PH group in contrast to the non-PH group (*P* < 0.05), and showed positive correlations with the parameters of RHC testing (mPAP, DPAP, SPAP) (*r* ranged 0.586~0.752 for MPA, 0.527~0.640 for RPA, and 0.302~0.495 for LPA, all with *P* < 0.05). For the MPA and RPA, 3D parameters showed higher correlation coefficients compared to their one-dimensional and two-dimensional counterparts. The ROC analysis indicated that the V_MPA_ showed higher area under the curves (AUC) than the D_MPA_ and A_MPA_ without significance, and the V_RPA_ showed higher AUC than the D_RPA_ and A_RPA_ significantly (D_RPA_ vs. V_RPA_, Z = 2.029, *P* = 0.042; A_RPA_ vs. V_RPA_, Z = 2.119, *P* = 0.034). The DCA demonstrated that the three-dimensional parameters could provide great net benefit for MPA and RPA. The predictive equations for mPAP, DPAP, and SPAP were formulated as [8.178 + 0.0006 * V_MPA_], [1.418 + 0.0005 * V_MPA_], and [-11.137 + 0.0006*V_RPA_ + 1.259 * D_MPA_], respectively.

**Conclusion:**

The 3D volume measurement of the MPA and RPA based on CTPA images maybe more informative than the traditional diameter and cross-sectional area in predicting PH.

## Introduction

Pulmonary hypertension (PH) refers to a complication of cardiopulmonary disease and a pathophysiological disorder with a cut-off level of mean pulmonary arterial pressure (mPAP) > 20mmHg at resting in right heart catheterization (RHC) test [1]. PH affects approximately 1% of the global population, up to 10% of individuals older than 65 years [2]. The observed PH prevalence has doubled in the last 10 years and is currently 125 cases/million inhabitants in the UK [3]. Persistent elevated pulmonary pressure and increased right heart load can lead to irreversible remodeling of pulmonary vessels, and at least 50% of patients with right heart failure [4]. The survival rate of pulmonary arterial hypertension ranged between 68% and 93% at 1 year and 39% and 77% at 3 years [2]. PH, especially chronic thromboembolic PH, is likely underdiagnosed early [5]. Given these potentially severe outcomes, timely diagnostic procedures and early therapeutic intervention are critical for the prognosis of PH.

RHC is the gold standard for measuring PH and has a class 1 indication to confirm the diagnosis [1]. However, RHC is an invasive examination with potential complications, which is impractical for clinical studies involving large cohorts of subjects. Besides, RHC provides limited information regarding the underlying cause of PH [6, 7]. Computed tomography pulmonary angiography (CTPA), a non-invasive imaging modality, can provide a more detailed assessment of the pulmonary vasculature [8]. Thus, CTPA has become an integral investigation in the PH diagnostic pathway. In most previous studies, a main pulmonary artery (MPA) diameter > 29 mm has traditionally been used as a threshold above which PH is suggested [9], and the ratio of ascending aorta diameter to the MPA diameter has also been suggested as a specific finding [10–12]. However, such measurements are based on a one-dimensional view, leading to limited sensitivity and specificity caused by a single-point evaluation and the potential for inter-observer variability.

Therefore, in this study, taking advantage of the three-dimensional (3D) model segmentation of the pulmonary artery tree based on CTPA images, we conducted a comparison study among the one-, two- and three-dimensional measurements of the MPA, right pulmonary artery (RPA), and left pulmonary artery (LPA). Thus, we aimed to evaluate the diagnostic value of 3D volumetry of the central pulmonary arteries for predicting PH based on CTPA images.

## Methods

### Materials

The retrospective study was conducted in accordance with the Declaration of Helsinki (as revised in 2013). Approval was granted by the ethics committee of Xi’an Jiaotong University, and all patients were fully informed of the nature of the study and the written consent for this retrospective analysis was waived.

**Fig. 1:**
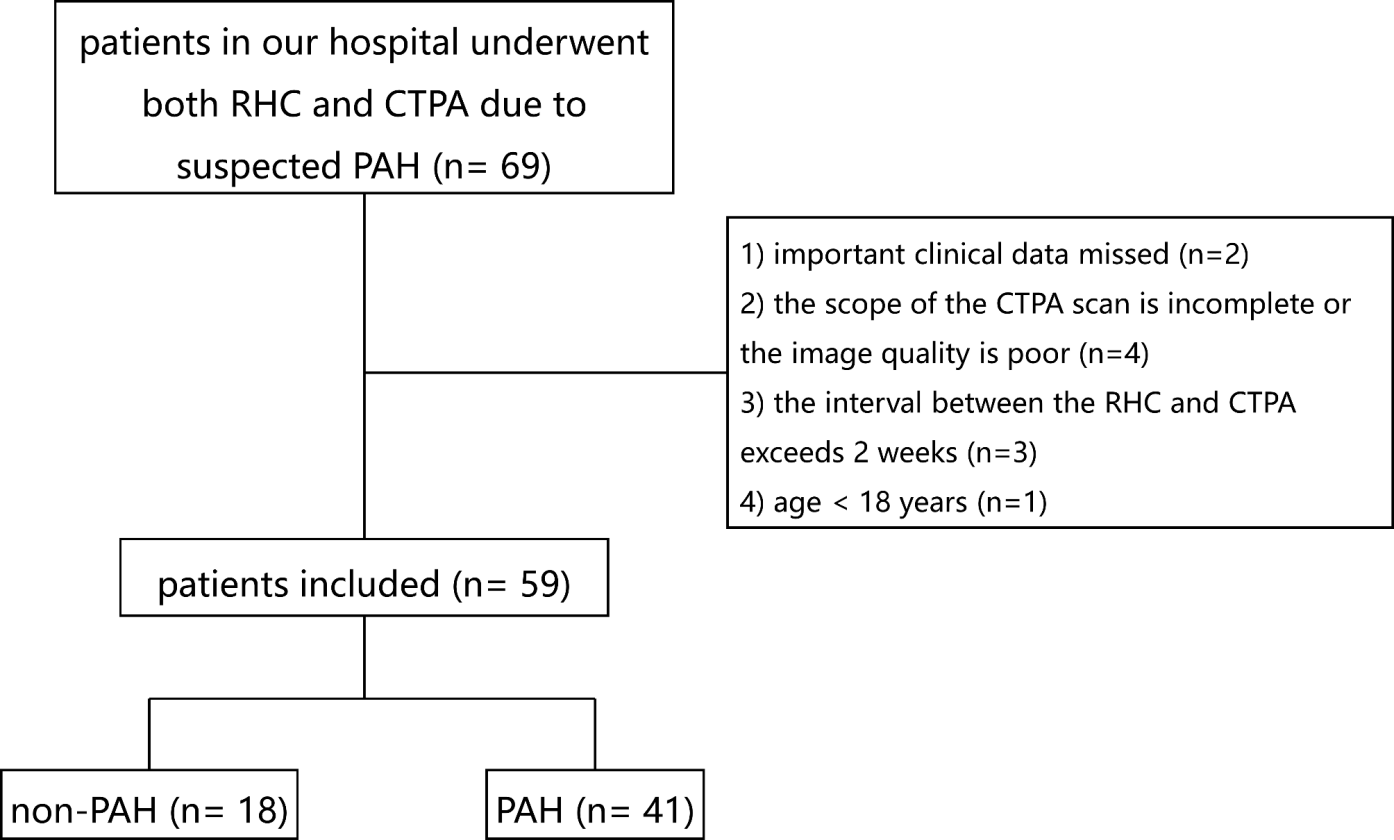
Flowchart of the study. (Abbreviations: PH, pulmonary hypertension; CTPA, computed tomography pulmonary angiography)

Records of 69 patients who underwent both RHC and CTPA due to suspected PH in the First Affiliated Hospital of Xi’an Jiaotong University from November 2013 to April 2021 were retrospectively collected. Inclusion criteria: (1) Patients who underwent an RHC examination; (2) CTPA imaging was conducted before the RHC examination; (3) CTPA images are stored in DICOM format. Exclusion criteria: (1) Absence of vital clinical information (n = 2); (2) Incomplete CTPA scan of the bilateral lungs or poor image quality (n = 4); (3) A time interval between RHC and CTPA exceeds 2 weeks (n = 3); (4) Patients aged below 18 years-old (n = 1). Following these criteria, 59 patients were selected for the study, including 18 males and 41 females, with a median age of 47 years (25%~75%, 33~61 years), see Fig. 1.

### Methods

#### RHC

RHC was performed with an 8 French introducer sheath placed in the right internal jugular vein under ultrasound guidance after local anesthetics. Then, a 7 French catheter (Swan-Ganz, Edwards Lifesciences) was placed into the main pulmonary artery for the measurement of mPAP, systolic pulmonary arterial pressure (SPAP), and diastolic pulmonary arterial pressure (DPAP). Two interventional physicians with more than 5 years of experience in RHC performed the procedures. PH is defined by a mean pulmonary arterial pressure (mPAP) > 20 mmHg at rest in the RHC testing.

#### CTPA imaging

All the CTPA images were performed on a 256-detector CT (Brilliance iCT 256, Philips Health Systems, Amsterdam, Netherlands) with patients in the supine position and breath holding. The scan scope was set from the apex to the base of the lungs. The scan parameters were as follows: area, 256 × 0.625 mm; tube voltage, 120 kV; automatic tube current technology, 59 to 189 mA (based on the patient’s weight); rotation time, 0.28 s; and pitch, 0.992:1.

The contrast agent (Iopromide, Bayer, Guangzhou, China, 370 mg iodine per milliliter, 50mL) was administered intravenously for all examinations. The injection rate was 5 ml/s in general and can be slowed down to 4.5mL/s for the elderly and low body mass patients. After contrast administration, a 40 mL saline chaser bolus was injected at the same flow rate. The scan was triggered automatically when the CT value of the right atrium (close to the inferior vena cava entrance) was larger than 120 Hounsfield Units (HU). All data were reconstructed using a standard reconstruction kernel. The field of view was 350 mm. The reconstruction matrix was 512 × 512. The slice thickness and the spacing between slices were 0.90 mm and 0.45 mm, respectively.

#### CTPA image evaluation

**Fig. 2:**
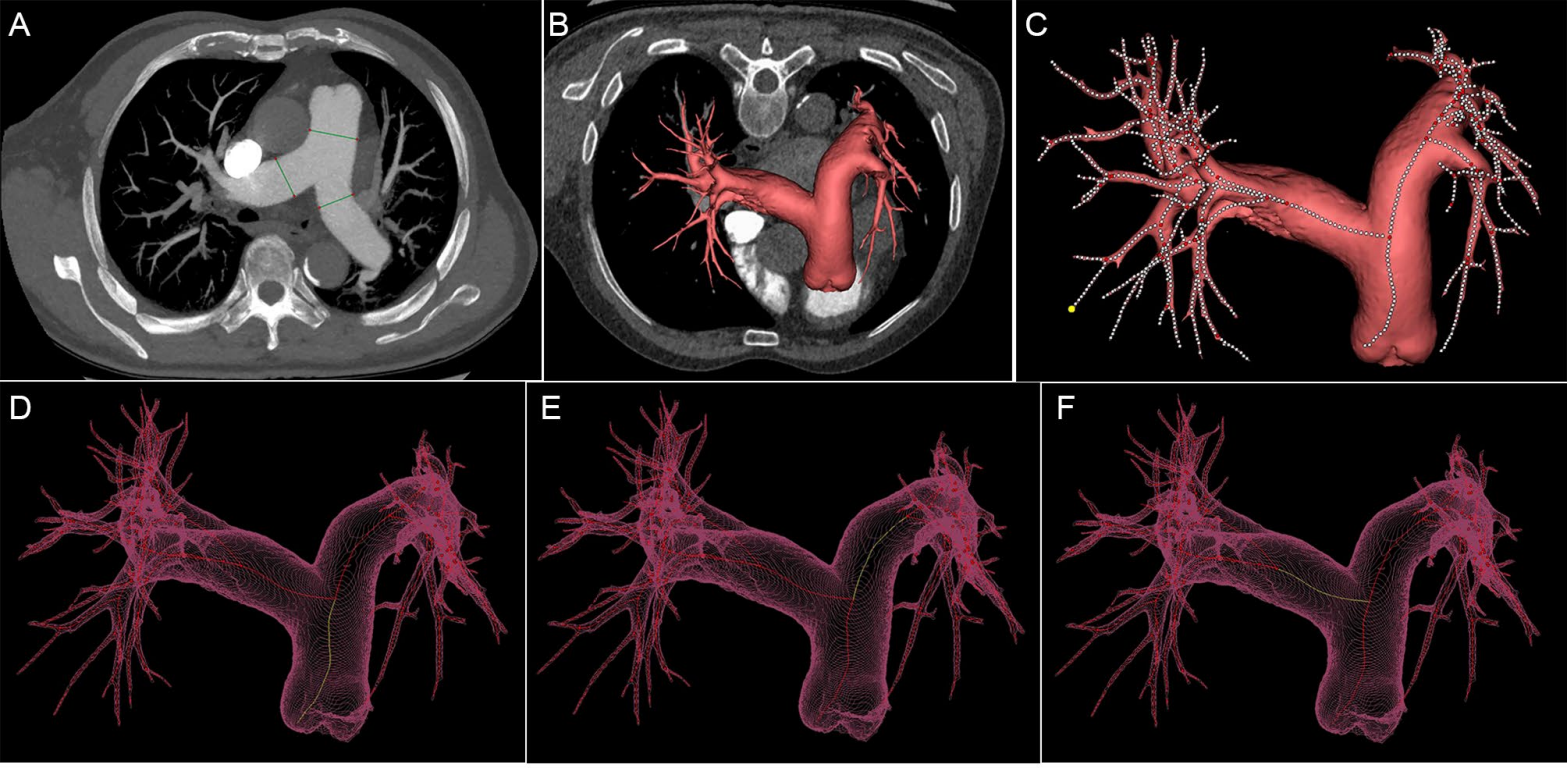
The measurement of pulmonary arteries on CTPA images. A patient was diagnosed with PH. The diameters were measured using the maximum density projection with a slice thickness of 10 mm at the bifurcation of the left and right pulmonary arteries on an axial slice (**2A**), where the D_MPA_, D_RPA_, and D_LPA_ were 40.50 mm, 23.30 mm, 26.90 mm. The 3D model was segmented using the software (**2B**), and the centerline was fitted (**2C**). The centerlines of MPA (**2D**), RPA (**2E**), LPA (**2F**) were marked. The A_MPA_, A_RPA_, and A_LPA_ were 1127.76 mm^2^, 582.59 mm^2^, 465.88mm^2^, and the V_MPA_, V_RPA_, and V_LPA_ were 70680.46 mm^3^, 44664.05 mm^3^, 47667.77 mm^3^. (Abbreviations: D_MPA_, the diameter of main pulmonary artery; A_MPA_, the cross-sectional area of main pulmonary artery; V_MPA_, the volume of the main pulmonary artery; D_RPA_, the diameter of the right pulmonary artery; A_RPA_, the cross-sectional area of the right pulmonary artery; V_RPA,_ the volume of right pulmonary artery; D_LPA_, diameter of left pulmonary artery; A_LPA_, cross-sectional area of left pulmonary artery; V_LPA_, the volume of the left pulmonary artery)

An experienced radiologist (WW.Z.) with ten years of experience in cardiovascular CTA reviewed the CTPA images for one-dimension MPA, RPA, and LPA measurements. Maximum density projection with a slice thickness of 10 mm was used, and the measurements were made at the 1.5 cm distally to the bifurcation of the left and right pulmonary arteries, perpendicular to its long axis, on an axial slice [13] (Fig. 2A). Measurements were avoided near the pulmonary valve or the bifurcation to ensure accuracy. The diameter of the MPA (D_MPA_), RPA (D_RPA_), and LPA (D_LPA_) was reported in millimeters (mm).

The Mimics 3D program (version 19.0, *Materialise*, Leuven, Belgium) was used to construct a 3D model of the central pulmonary arteries based on the DICOM files by another radiologist (C.S.) with 10 years of experience in cardiovascular CTA. Masking was performed using the threshold of a minimum value of − 200 HU and a maximum value of 500 HU to reconstruct the pulmonary artery initially. Afterward, the non-relevant structures, such as bones and tissues outside the pulmonary, were cut by the menu of free hand. Next, the dynamic region-growing method was applied using a seed placed within the MPA to remove scattered branches. The manual correction (by two 10-year experienced radiologist, NL.D. and HH.H) was allowed when the segmentation was not satisfied (Fig. 2B). All readers were blinded to the reported clinical diagnosis. The centerline of the 3D object was fitted with the smoothing factor of 0.5, resolving resolution of 1 mm, and distance between two successive control points of 1 mm (Fig. 2C).

The cross-sectional area perpendicular to the vessel center line of each control point was calculated automatically. The slices of interest of MPA, RPA, and LPA were selected (by W. Z), avoiding the pulmonary valve or the bifurcation. Thus, the cross-sectional area of the MPA (A_MPA_), RPA (A_RPA_), and LPA (A_LPA_) were recorded in square millimeters (mm^2^). The volume of the MPA (V_MPA_), RPA (V_RPA_), and LPA (V_LPA_) were determined by summing the cross-sectional areas of each slice within their respective arteries, and the volumes were recorded as cubic millimeters (mm^3^) (Fig. 2D and E, and 2F).

#### Statistical analysis

The statistical analysis was performed using SPSS 25.0 software (IBM Corp., Armonk, NY, USA). Descriptive statistics were expressed as mean ± standard deviation (SD) or median with interquartile range for continuous variables and as frequency and percentages for categorical variables. To compare differences between groups, the Student t-test or Wilcoxon rank-sum test was employed for continuous variables, and Fisher’s exact test was applied for categorical variables as appropriate. Shapiro-Wilk test was used for the normality test of the continuous variables.

The parameters of CTPA (D_MPA_, D_RPA_, D_LPA_, A_MPA_, A_RPA_, A_LPA,_ V_MPA_, V_RPA_, and V_LPA_) were correlated with the parameters of RHC (mPAP, DPAP, and SPAP) using the Spearman or Pearson correlation test, depending on the data distribution. ROC curves were used to determine the optimal cutpoints of the CTPA parameters for predicting PH. Sensitivity, specificity, positive predictive value (PPV), negative predictive value (NPV), accuracy, and the area under the curve (AUC) were calculated. The comparisons of the AUCs were conducted using the *Delong* test by MedCalc® Statistical Software version 22.014 (MedCalc Software Ltd, Ostend, Belgium; https://www.medcalc.org; 2023). Decision curve analysis (DCA) were used to evaluate thenet benefit of three-dimensional parameters for predicting PH using R software (version 4.3.2). Multiple linear regression models with a forward step were used to incorporate all statistically significant CTPA parameters, select the most important CTPA parameters, and fit a straight line with a 95% confidence interval (CI) that predicts the pulmonary arterial pressures. A *P* value < 0.05 was considered significant for all statistical analyses.

## Results

### Basic clinical characteristics of two groups

A total of 59 patients (42 females, 17 males) were included, with 18 cases (30.5%) in the non-PH and 41 cases (69.5%) in the PH group. In the PH group, there was a significant increase in the respiratory rate and higher occurrences of dyspnea and dizziness compared to the non-PH group (*P* < 0.05). Furthermore, patients in the non-PH group exhibited a higher frequency of accompanying congenital heart disease, while a history of acute pulmonary embolism was more prevalent in the PH group (*P* < 0.05), see Table 1.

**Table 1:** Clinical characteristics of the patients in two groups

	non-PH (n = 18)	PH (n = 41)	*T/Z/χ* ^*2*^	*P*
Age (year)	48.22 ± 17.96	47.65 ± 14.91	0.128†	0.898
Sex (male/female)	7 / 11	10 / 31	1.282§	0.258
**Signs**				
aortic diastolic blood pressure (mmHg)	75.78 ± 11.09	77.67 ± 10.97	-0.614†	0.542
aortic systolic blood pressure (mmHg)	126.28 ± 21.43	116.98 ± 16.42	1.840†	0.071
respiratory rate (times/min)	18 (18~20)	20.00 (19~20)	-2.083‡	**0.037**
pulse (times/min)	80 (71.5~84)	82.5 (72.75~91.75)	-1.401‡	0.161
**Symptoms**				
dyspnea (%)	4 (22.2%)	36 (83.6%)	24.642§	**< 0.001**
fever (%)	1 (5.6%)	0 (0%)	-	0.295
dizzy (%)	8 (44.4%)	0 (0%)	-	**< 0.001**
edema of both lower limbs (%)	1 (5.6%)	1 (2.3%)	-	0.507
chest pain (%)	1 (5.6%)	1 (2.3%)	-	0.507
palpitation (%)	0 (0%)	2 (4.7%)	-	1.000
syncope (%)	1 (5.6%)	2 (4.7%)	-	1.000
**Medical history**				
congenital heart disease (%)	12 (66.7%)	10 (24.4%)	9.560§	**0.002**
acute pulmonary embolism (%)	2 (11.1%)	20 (48.7%)	-	**0.008**
chronic obstructive lung disease (%)	0 (0%)	3 (7.0%)	-	0.548
coronary heart disease (%)	3 (16.7%)	11(26.8%)	-	0.516
rheumatic disease (%)	0 (0%)	1 (2.3%)	-	1.000
**Group according to 2022 ESC/ERS guidelines**				
Group 1 (male/female)	NA	11(1/10)	NA	NA
Group 2 (male/female)	NA	8(3/5)	NA	NA
Group 3 (male/female)	NA	2(0/2)	NA	NA
Group 4 (male/female)	NA	17(4/13)	NA	NA
Group 5 (male/female)	NA	3(2/1)^a^	NA	NA

Note: †, *t-test*; ‡, *Z* test; §, *χ*^*2*^ test; --, Fisher exact probability test; a, the three patients in group 5 had both left heart disease and chronic pulmonary embolism PH

Abbreviations: PH, pulmonary hypertension; NA, not appliable

### Comparison of RHC and CTPA parameters in two groups

The parameters of the RHC (mPAP, SPAP, and DPAP) and CTPA (D_MPA_, D_RPA_, D_LPA_, A_MPA_, A_RPA_, A_LPA,_ V_MPA_, V_RPA_, V_LPA_) were significantly higher in the PH group than in the non-PH group (*P* < 0.05), see Table 2.

**Table 2:** The pulmonary arterial pressures and measurements of the pulmonary arteries on CTPA images of the patients in two groups

	non-PH (*n* = 18)	PH (*n* = 41)	*T/Z*	*P*
**RHC**				
SPAP (mmHg)	26.00 (24~29)	72.22 ± 19.73	-5.940‡	**< 0.001**
mPAP (mmHg)	18 (16~19)	26 (20~35)	-5.711‡	**< 0.001**
DPAP (mmHg)	10 (8.5~11)	27.85 ± 10.77	-6.079‡	**< 0.001**
**CTPA**				
D_MPA_ (mm)	25.93 ± 4.68	36.01 ± 6.36	-5.901†	**< 0.001**
A_MPA_ (mm^2^)	601.57 ± 184.37	1046.88 ± 278.94	-6.285†	**< 0.001**
A_MPA_ (mm^3^)	25751.64 ± 7154.24	55557.10 ± 18449.48	-8.511†	**< 0.001**
D_RPA_ (mm)	19.37 ± 3.32	24.37 ± 4.15	-4.498†	**< 0.001**
A_RPA_ (mm^2^)	348.58 ± 114.38	536.47 ± 176.59	-4.041†	**< 0.001**
V_RPA_ (mm^3^)	15594.91(12662.13~29474.24)	48454.73 ± 19118.22	-4.893‡	**< 0.001**
D_LPA_ (mm)	17.51 ± 3.47	21.68 ± 4.36	-3.603†	**0.001**
A_LPA_ (mm^2^)	314.36 ± 75.18	459.79 ± 182.64	-4.075 †	**< 0.001**
V_LPA_ (mm^3^)	23118.99 ± 13552.82	37963.42 ± 16972.93	-3.073†	**0.003**

Note: †, *t-test*; ‡, *Z* test;

Abbreviations: PH, pulmonary hypertension; RHC, right heart catheterization; SPAP, systolic pulmonary artery pressure; mPAP, mean pulmonary arterial pressure; DPAP, diastolic pulmonary arterial pressure; CTPA, computed tomography pulmonary angiography; D_MPA_, diameter of main pulmonary artery; A_MPA_, cross-sectional area of main pulmonary artery; V_MPA_, volume of main pulmonary artery; D_RPA_, diameter of right pulmonary artery; A_RPA_, cross-sectional area of right pulmonary artery; V_RPA,_ volume of right pulmonary artery; D_LPA_, diameter of left pulmonary artery; A_LPA_, cross-sectional area of left pulmonary artery; V_LPA_, volume of left pulmonary artery

### Correlation analysis

**Fig. 3:**
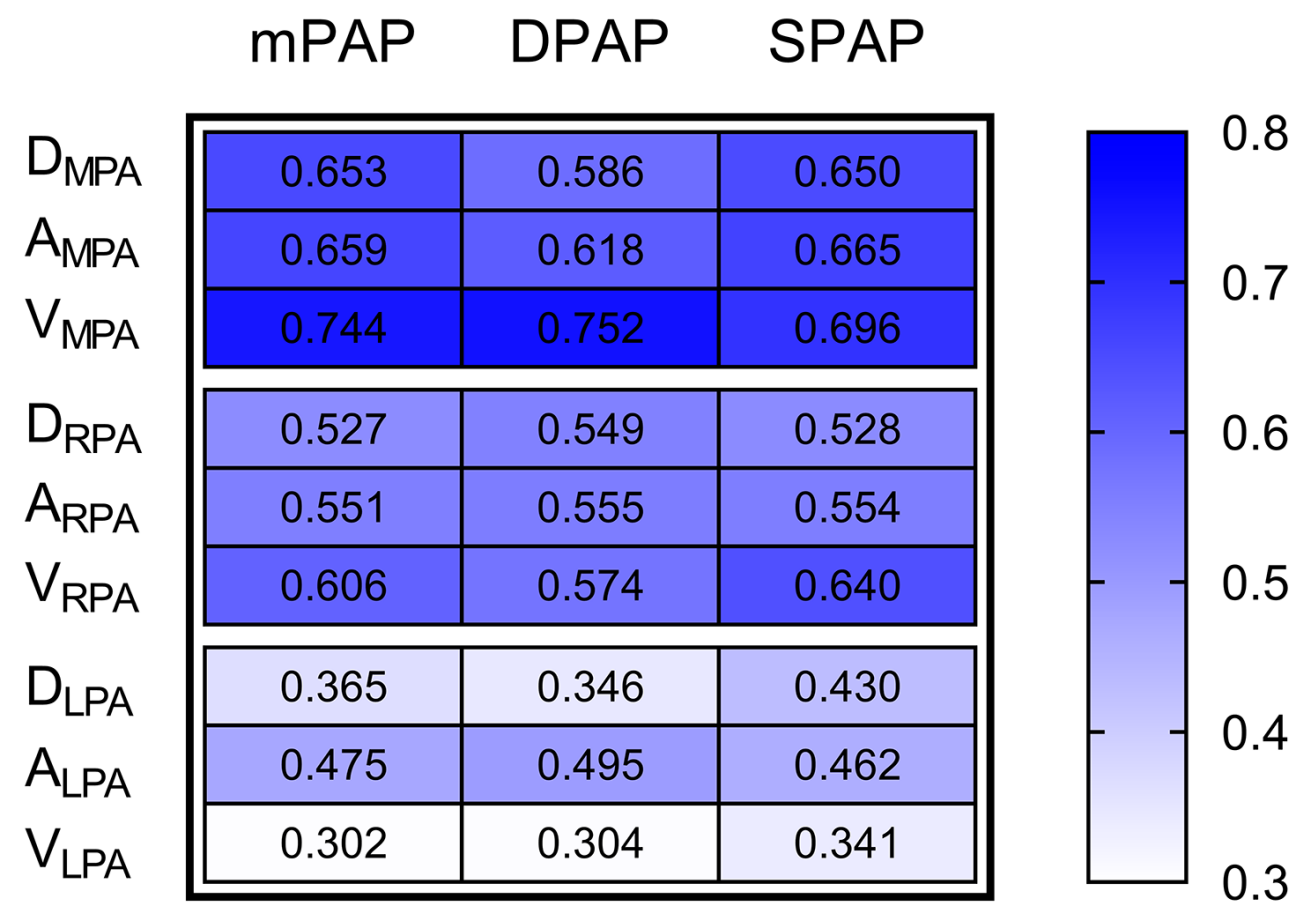
The heatmap of the correlation coefficient between the measurements of RHC and the CTPA results for all patients (Abbreviations: RHC, right heart catheterization; mPAP, mean pulmonary arterial pressure; DPAP, diastolic pulmonary arterial pressure; SPAP, systolic pulmonary artery pressure; CTPA, computed tomography pulmonary angiography; D_MPA_, diameter of main pulmonary artery; A_MPA_, cross-sectional area of main pulmonary artery; V_MPA_, volume of main pulmonary artery; D_RPA_, diameter of right pulmonary artery; A_RPA_, cross-sectional area of right pulmonary artery; V_RPA,_ volume of right pulmonary artery; D_LPA_, diameter of left pulmonary artery; A_LPA_, cross-sectional area of left pulmonary artery; V_LPA_, volume of left pulmonary artery)

The one-dimensional (D_MPA_, D_RPA_, D_LPA_), two-dimensional (A_MPA_, A_RPA_, A_LPA_), and three-dimensional (V_MPA_, V_RPA_, V_LPA_) measurements on CTPA images exhibited significant positive correlations with the pulmonary artery pressures (mPAP, DPAP, SPAP) obtained from RHC, and all *P* < 0.05. From the heatmap of the correlation coefficients between the measurements of RHC and the CTPA (Fig. 3), we can read that, for the MPA and RPA, the correlation coefficients of the three-dimensional CTPA parameters were higher than the one-dimensional and two-dimensional parameters. However, for the LPA, the correlation coefficients of the three-dimensional parameters were lower than the one-dimensional and two-dimensional parameters.

### The ROC analysis of the diagnostic efficacy of CTPA parameters in predicting PH

**Fig. 4:**
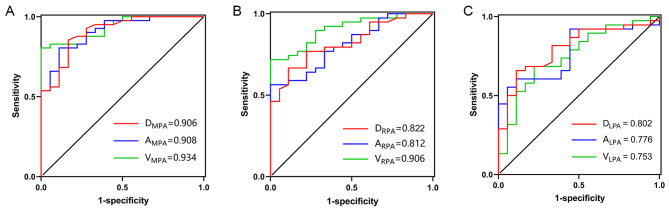
ROC curve comparisons among one-, two- and three-dimensional parameters of the main (**4A**), right (**4B**), and left (**4C**) pulmonary artery in predicting PH. The comparison of the ROC curves in predicting PH among the volumes (the green lines), cross-sectional areas (the blue lines), and the diameters (the red lines) of the MPA (**4A**), RPA (**4B**), and LPA (**4C**). (Abbreviations: ROC, receiver operating characteristic; AUC, area under the curve; D_MPA_, diameter of main pulmonary artery; A_MPA_, cross-sectional area of main pulmonary artery; V_MPA_, volume of main pulmonary artery; D_RPA_, diameter of right pulmonary artery; A_RPA_, cross-sectional area of right pulmonary artery; V_RPA,_ volume of right pulmonary artery; D_LPA_, diameter of left pulmonary artery; A_LPA_, cross-sectional area of left pulmonary artery; V_LPA_, volume of left pulmonary artery)

For the MPA, the volume showed higher AUC than the diameter and sectional area (see Table 3; Fig. 4A), but the Delong tests showed no significant difference among the three ROC curves (D_MPA_ vs. A_MPA_, Z = 0.092, *P* = 0.927; D_MPA_ vs. V_MPA_, Z = 0.788, *P* = 0.431; A_MPA_ vs. V_MPA_, Z = 0.887, *P* = 0.375).

**Table 3:** ROC curve for the identification of PH based on CTPA measurements

	cutoff value	AUC	95% CI	sensitivity	specificity	PPV	NPV	Accuracy
D_MPA_ (mm)	29.300	0.906	0.832~0.990	0.853	0.833	0.904	0.764	0.687
A_MPA_ (mm^2^)	803.005	0.908	0.827~0.984	0.805	0.889	0.904	0.764	0.694
V_MPA_ (mm^3^)	38853.430	0.934	0.887~0.999	0.805	1.000	0.881	0.722	0.805
D_RPA_ (mm)	22.900	0.822	0.713~0.932	0.667	0.889	0.761	0.583	0.556
A_RPA_ (mm^2^)	499.998	0.812	0.705~0.928	0.564	1.000	0.795	0.642	0.564
V_RPA_ (mm^3^)	39564.981	0.906	0.839~0.985	0.718	1.000	0.860	0.706	0.718
D_LPA_ (mm)	20.750	0.802	0.681~0.921	0.657	0.889	0.734	0.555	0.546
A_LPA_ (mm^2^)	417.252	0.776	0.668~0.909	0.552	0.944	0.705	0.428	0.517
V_LPA_ (mm^3^)	27277.279	0.753	0.629~0.898	0.684	0.778	0.782	0.667	0.462

Abbreviations: ROC, receiver operator characteristic; AUC, areas under the curve; PPV, positive predictive value; NPV, negative predictive value; D_MPA_, diameter of main pulmonary artery; A_MPA_, cross-sectional area of main pulmonary artery; V_MPA_, volume of main pulmonary artery; D_RPA_, diameter of right pulmonary artery; A_RPA_, cross-sectional area of right pulmonary artery; V_RPA,_ volume of right pulmonary artery; D_LPA_, diameter of left pulmonary artery; A_LPA_, cross-sectional area of left pulmonary artery; V_LPA_, volume of left pulmonary artery; CI, confidential interval

For the RPA, the volume showed higher accuracy than the diameter and cross-sectional area (see Table 3; Fig. 4B); meantime, the Delong tests showed that volume had significantly a greater area under the curve in predicting PH than the counterparts of diameter and the sectional area (D_RPA_ vs. V_RPA_, Z = 2.029, *P* = 0.042; A_RPA_ vs. V_RPA_, Z = 2.119, *P* = 0.034).

The volume showed lower accuracy for the LPA than the diameter and sectional area (see Table 3; Fig. 4C). The Delong test showed no significant difference among the three curves (D_LPA_ vs. A_LPA_, Z = 0.286, *P* = 0.775; D_LPA_ vs. V_LPA_, Z = 0.379, *P* = 0.705; A_LPA_ vs. V_LPA_, Z = 0.206, *P* = 0.837).

**Fig. 5:**
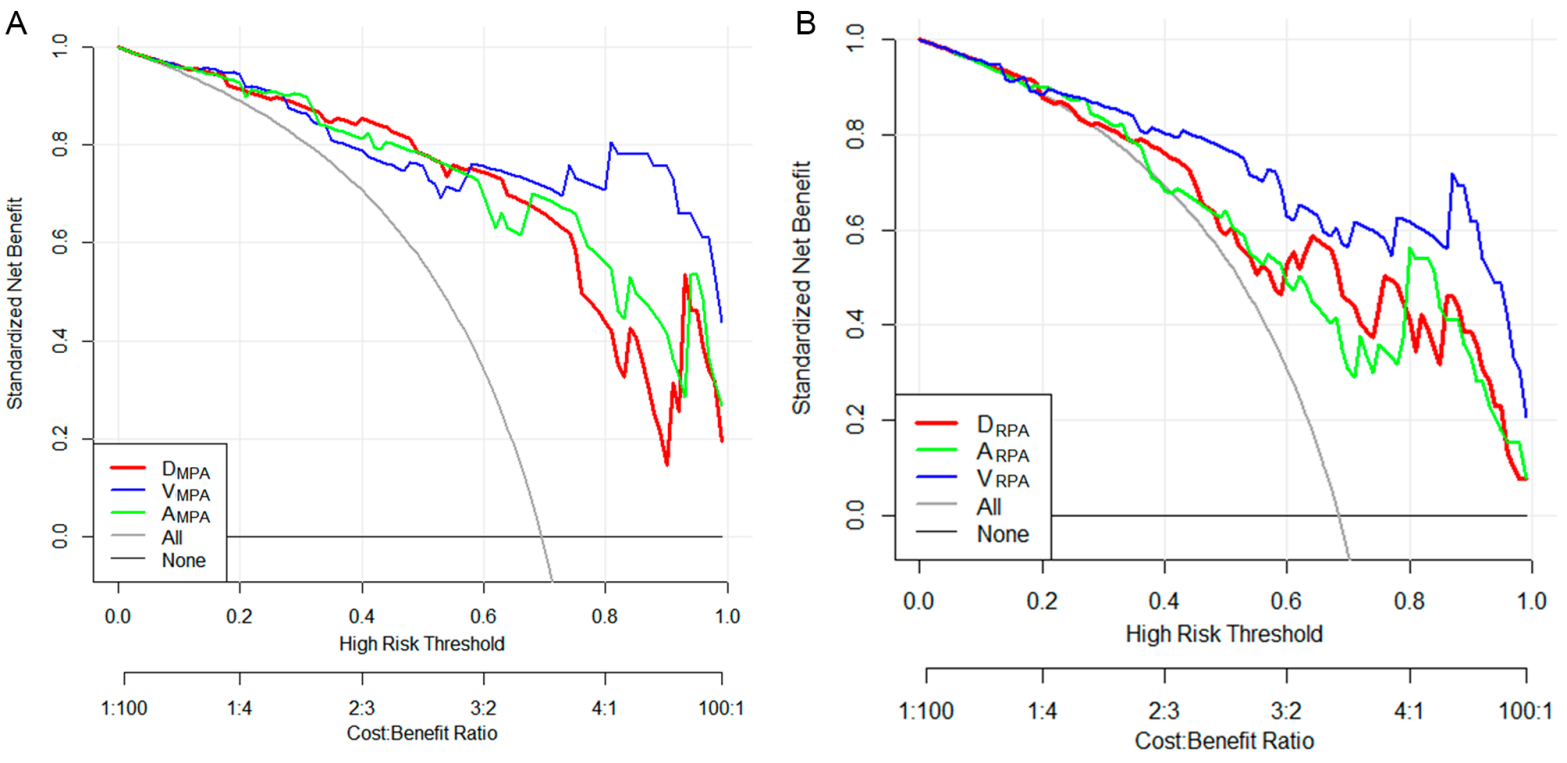
Decision curve analysis of the CTPA parameters for the MPA (**A**) and RPA (**B**) in the estimation of PH. In the Figure, the *x*-axis is the threshold probability, and the *y*-axis measures the net benefit. The red line represents the one-dimensional, the green line represents the two-dimensional, and the blue line represents the three-dimensional parameters of the MPA and RPA. (Abbreviations: CTPA, computed tomography pulmonary angiography; PH, pulmonary hypertension; D_MPA_, diameter of main pulmonary artery; A_MPA_, cross-sectional area of main pulmonary artery; V_MPA_, volume of main pulmonary artery; D_RPA_, diameter of right pulmonary artery; A_RPA_, cross-sectional area of right pulmonary artery; V_RPA,_ volume of right pulmonary artery)

The DCA demonstrated that the three-dimensional parameters could provide great net benefit for MPA and RPA, see Fig. 5.

### Linear regression model for the prediction of PH

In the multiple linear regression model, the predictive equation of mPAP, DPAP, and SPAP were as follows:

predicted mPAP = 8.178 + 0.0006 * V_MPA_.

predicted DPAP = 1.418 + 0.0005 * V_MPA_.

predicted SPAP = -11.137 + 0.0006 * V_RPA_ + 1.259 * D_MPA_.

**Fig. 6:**
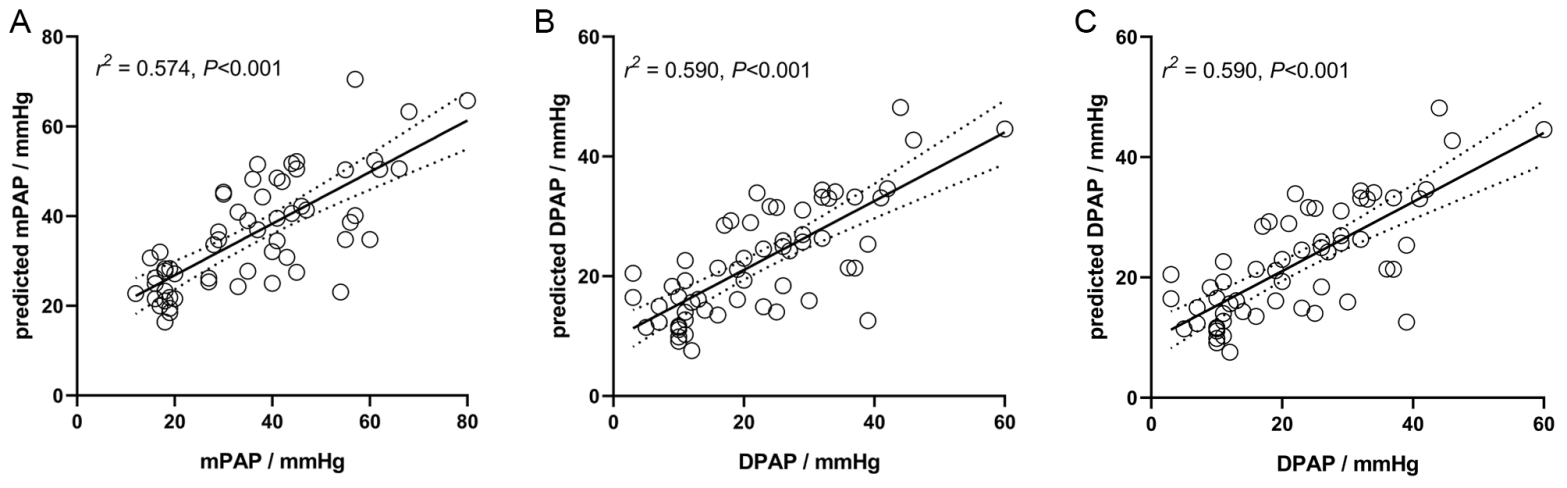
The scatter plot of the predicted PAP and the real-world PAP. **(**Abbreviations: mPAP, mean pulmonary arterial pressure; DPAP, diastolic pulmonary arterial pressure; SPAP, systolic pulmonary artery pressure)

The prediction of the mPAP, DPAP, and SPAP showed good Goodness of fit with its true values, see Fig. 6.

## Discussion

With the continuous development of CT technology, CTPA is frequently clinically indicated and performed to confirm PH diagnosis [14]. Previous studies on the role of CTPA in PH mainly focused on measuring pulmonary artery diameter and the ratio of the pulmonary artery to aortic diameter, which showed particular limitations in terms of sensitivity and negative predictive values [9, 12, 15–17]. This study compared the one-, two- and three-dimensional parameters of the central pulmonary arteries based on CTPA images in evaluating PH. The results showed that the volume of MPA and RPA had advantages in predicting the PH compared with the one- and two- dimensional parameters. Although the diameter, cross-sectional area, and volume of the central pulmonary arteries increased significantly in PH patients (*P* < 0.05), the volume of MPA and RPA had a higher correlation with the mPAP, SPSP and DPAP than the diameter and the cross-sectional area (*P* < 0.05). The volume of RPA performed significantly better than the diameter and the cross-sectional areaof RPA.

The patients included in this study were younger than the previous study (with the median age of 65–69 years-old) [14], mainly due to the higher proportion of group 1 PH, which is often seen in young females [1]. And females in the PH group was about 3 times than the males. These findings are well in accordance with previous studies investigating pulmonary artery diameters. Fabian Rengier measured the volume of MPA, RPA, and LPA on the magnetic resonance angiography images, and the pulmonary artery volume showed higher sensitivity and specificity for predicting PH compared to pulmonary artery diameters manually measured on axial reconstructions [18]. Melzig, C. also measured the volume of MPA, RPA, and LPA with CTPA images, the volume of MPA showed a strong correlation with mPAP (*r* = 0.76, *P* < 0.001), and the AUC of the MPA for the prediction of PH were 0.90 [10]. In our result, the correlations of the V_MPA_ with mPAP, SPAP, and DPAP were 0.744, 0.752, and 0.696 (*P* < 0.05), and the AUC of MPA for the PH prediction was 0.934, which was a little higher than Melzig’s results. Thus, compared with the diameter of the pulmonary artery only at an interested slice, quantitative measurement of 3D geometric changes of the entire blood vessels can improve the reliability of PH diagnosis.

In addition, this study found that the correlation coefficient of the volume of LPA was lower than that of the diameter and area of LPA, this is also in accordance with Melzig’s results [11]. This lower correlation can be explained by the more significant variability of LPA among patients, and the volume measurement of the LPA magnified this kind of variability.

This study also derived the linear regression models for the prediction of PAPs, V_MPA_ was selected as the most discriminative factor in the evaluation of mPAP and DPAP, with a goodness of fit of 0.574, and 0.590, respectively. At the same time, V_RPA_ and D_MPA_ were selected as predictors of SPAP, with a goodness of fit of 0.583.

To our knowledge, this is the first study that evaluated the feasibility of the quantitative volume measurement of central pulmonary arteries in the PH prediction since the update of the cutoff point of PH to 20mmHg. With the advanced image post-processing technology, the pulmonary artery segmentation would be more accessible, and the volume measurement of the central pulmonary artery would improve the evaluation of the pulmonary arterial pressure.

This study has limitations. First, the retrospective nature of this study may introduce selection bias. Compared with the pulmonary hypertension group, the control group had a higher rate of congenital heart disease, which may have a particular impact on the result, and prospective studies are needed to confirm these findings. Second, the sample size of this study is small. PH patients were classified into five groups according to the different causes [1], the small sample size is not enough for the subgroup analysis. Third, the pulmonary artery was not corrected for the body surface area due to missing clinical data in more than 50% of patients. However the age and sex ratio of the two groups were balanced, and the comparison of the one-, two-, and three-dimensional measurements was conducted in pairs, thus not affect the usefulness of the volume measurement of central pulmonary arteries in the PH prediction.

In summary, compared with the commonly used measurement of pulmonary artery diameter and area, the volume measurement of the central pulmonary artery based on CTPA can deliver higher accuracy for of diagnosing PH.

## Data Availability

Data and materials generated or analyzed during the study are available from the corresponding author by request.

## Abbreviations

AUC: area under the curve
A_MPA_: cross-sectional area of the main pulmonary artery
A_RPA_: cross-sectional area of right pulmonary artery
A_LPA_: cross-sectional area of left pulmonary artery
CI: confidential interval
CTPA: computer tomography pulmonary angiography
DPAP: diastolic pulmonary artery pressure
D_MPA_: diameters of main pulmonary artery
D_RPA_: diameter of right pulmonary artery
D_LPA_: diameter of left pulmonary artery
LPA: left pulmonary artery
MPA: main pulmonary artery
mPAP: mean pulmonary artery pressure
PH: pulmonary hypertension
RPA: right pulmonary artery
RHC: right heart catheterization
SPAP: systolic pulmonary artery pressure
V_MPA_: volume of main pulmonary artery
V_RPA_: volume of right pulmonary artery
V_LPA_: volume of left pulmonary artery
